# The p85 Regulatory Subunit of PI3K Mediates cAMP-PKA and Insulin Biological Effects on MCF-7 Cell Growth and Motility

**DOI:** 10.1155/2014/565839

**Published:** 2014-07-09

**Authors:** E. Di Zazzo, A. Feola, C. Zuchegna, A. Romano, C. F. Donini, S. Bartollino, C. Costagliola, R. Frunzio, P. Laccetti, M. Di Domenico, A. Porcellini

**Affiliations:** ^1^Department of Medicine and Health Sciences, University of Molise, Campobasso, Italy; ^2^Department of Biology, University Federico II of Naples, Naples, Italy; ^3^Department of Biochemistry, Biophysics and General Pathology, Second University of Naples, Italy; ^4^Department of Experimental Medicine, Sapienza University of Rome, Rome, Italy; ^5^Sbarro Institute for Cancer Research and Molecular Medicine, Center for Biotechnology, Temple University, Philadelphia, PA, USA

## Abstract

Recent studies have shown that hyperinsulinemia may increase the cancer risk. Moreover, many tumors demonstrate an increased activation of IR signaling pathways. Phosphatidylinositol 3-kinase (PI3K) is necessary for insulin action. In epithelial cells, which do not express GLUT4 and gluconeogenic enzymes, insulin-mediated PI3K activation regulates cell survival, growth, and motility. Although the involvement of the regulatory subunit of PI3K (p85*α* ^PI3K^) in insulin signal transduction has been extensively studied, the function of its N-terminus remains elusive. It has been identified as a serine (S83) in the p85*α* ^PI3K^ that is phosphorylated by protein kinase A (PKA). To determine the molecular mechanism linking PKA to insulin-mediated PI3K activation, we used p85*α* ^PI3K^ mutated forms to prevent phosphorylation (p85A) or to mimic the phosphorylated residue (p85D). We demonstrated that phosphorylation of p85*α* ^PI3K^S83 modulates the formation of the p85*α* ^PI3K^/IRS-1 complex and its subcellular localization influencing the kinetics of the insulin signaling both on MAPK-ERK and AKT pathways. Furthermore, the p85*α* ^PI3K^S83 phosphorylation plays a central role in the control of insulin-mediated cell proliferation, cell migration, and adhesion. This study highlights the p85*α* ^PI3K^S83 role as a key regulator of cell proliferation and motility induced by insulin in MCF-7 cells breast cancer model.

## 1. Introduction

Over the past decade several studies have shown that insulin therapy may increase the cancer risk [1, 2]. In addition, several epidemiological studies have reported that obese people and those with type 2 diabetes have a higher risk of developing cancer [3, 4]. Insulin resistance that occurs in obese people and those with type 2 diabetes induces a compensatory increase in insulin levels [5]. Some cancer cells have increased insulin receptor (IR) content, and in the setting of hyperinsulinemia, certain tumors may demonstrate increased activation of IR signaling pathways [6, 7]. Hyperinsulinemia, in a nonobese mouse model of type 2 diabetes, has been reported to lead to increased mammary tumor growth [7]. Again, blocking the insulin receptor (IR) tyrosine kinase, using the inhibitor BMS-536924, reduced tumor growth in these mice [7]. Moreover, reduction of circulating insulin levels using a beta 3-adrenergic receptor agonist (CL-316243) or downregulation of the IR in cancer cells and xenografts decreased tumor growth [8] and reduced cell proliferation and metastasis [9].

The phosphoinositide 3-kinase (PI3K) signaling pathway critically regulates cell growth and survival [10, 11]. Class IA phosphoinositide 3-kinase (PI3K) is a heterodimeric enzyme composed of a catalytic subunit (p110) and a regulatory subunit (p85). The regulatory p85 subunit (p85*α* ^PI3K^) consists of several domains including the SH3 domain, two proline rich fragments, and two SH2 domains separated by the inter-SH2 (iSH2) sequence [12]. The p85 adaptor protein employs the iSH2 region to bind the p110 catalytic subunit and employs SH3 and SH2 domains, phosphotyrosine residues, and proline-rich motifs to interact with activated tyrosine kinases [13, 14], adaptor proteins, estrogen and androgen receptors, retinoic acid receptor, and PKA [15–17]. The PI3K-Akt pathway is activated by binding of p85*α* ^PI3K^ to tyrosine-phosphorylated sites on growth factor receptors or on associated adaptor proteins such as IRS-1 (insulin receptor substrate 1) [18]. Protein kinase B (PKB), also called Akt, is a well-known target of PI3K and regulates multiple biological functions, including gene expression, cell cycle, survival, insulin-induced metabolic signals, endocytosis and vascular trafficking, cell transformation, and oncogenesis [19]. The ERK and Akt pathways work coordinately or synergically to promote cell growth and progression through the cell cycle [20]. The Akt pathway stimulates the uptake of glucose [21], whereas ERK activates immediate-early transcription factors including c-Fos and Ets-1. Because the ERK and Akt pathways are pleiotropic and interconnected, it is difficult to determine the distinct contribution of each to the overall proliferative response to growth factors. Nonetheless, both phosphorylated Akt and ERK (pAkt and pERK, resp.) are frequently used as readouts of proliferative or oncogenic signalling [22, 23].

PI3K plays a critical role in insulin signalling and the p85*α* ^PI3K^ exerts both positive and negative effects on the insulin transduction pathways [24, 25]. The activity of PI3K is necessary to elicit many of the effects of insulin on glucose and lipid metabolism, indicating that it is an essential downstream effector of insulin signalling. The principal mechanisms through which insulin activates PI3K appear to be via IRS-1/2 proteins [26]. PI3K interacts with IRS-1 and IRS-2 docking proteins and plays a key role in mediating insulin signal downstream from IRS-1 that results, for example, in MCF-7 cell line in cell cycle progression [22]. While necessary for the stability and membrane recruitment of the p110 catalytic subunit of PI3K, p85*α* ^PI3K^ represses the basal activity of p110 in the absence of growth factor stimulation. In some cell types p85 is in excess of p110, and free, monomeric p85*α* ^PI3K^ acts as a negative regulator of PI3K signalling [23, 27]. In its unbound form, p85*α* ^PI3K^ sequesters the adaptor protein IRS-1 forming cytosolic foci free of p110 and therefore limits the extent of PI3K signaling downstream of the insulin and IGF-1 receptors. The SH3 domain located in the N-terminal portion of p85*α* ^PI3K^ is responsible for the interaction with IRS-1 [28].

We have identified a serine (at codon 83) adjacent to N-terminus SH3 domain in the PI3K regulatory subunit p85*α* ^PI3K^ that is critical for cell cycle progression, cell survival, and migration.   p85*α* ^PI3K^Ser83 is phosphorylated by protein kinase A (PKA)* in vivo* and* in vitro*, influencing the ability of SH3 domain to interact with different partners [16, 17, 29].

The aim of this work was to analyze the role of the SH3 domain of p85*α* ^PI3K^ in insulin signalling, used as model the breast cancer cell line MCF-7. For this purpose, we tested whether phosphorylation of p85*α* ^PI3K^S83 modulates the ability of p85*α* ^PI3K^ to form complex with IRS-1 or IRS-2 and therefore influences the insulin signaling in MCF-7 cells. Furthermore, we analyzed the biological consequences of the expression of mutant versions of p85*α* ^PI3K^ in MCF-7 cells, focusing on the role of p85*α* ^PI3K^S83 in cell motility, growth, and survival.

## 2. Materials and Methods

### 2.1. Cell Cultures and Plasmid Transfections

MCF-7 cells were obtained from American Type Culture Collection (ATCC, Rockville, MD, USA) and cultured as previously described [16]. All experiments were performed in cells after a serum starvation for 6 h and growing for the time indicated in the legend of figures with 0.5% FCS and 2 mM L-glutamine DMEM (Lonza; Verviers, Belgium). For treatment MCF-7 cells were grown in the same medium supplemented with 10 nM insulin (Sigma-Aldrich, Co., St. Louis, MO, USA) with or without 10 *μ*g/mL PKA inhibitor P9115 (Sigma-Aldrich, Co., St. Louis, MO, USA) for 15 minutes.

Plasmids carrying bovine p8WT tagged with FLAG (pSG5-FLAG-p85 wt), or its S83 mutated forms, p85A (pSG5-FLAG-p85A) and p85D (pSG5-FLAG-p85 D), were obtained as previously described [16]. Transfections were performed using Lipofectamine-GIBCO BRL, Life Technologies (Rockville, MD, USA) following the manufacturer's instructions. In all transfections, pEGFPC3 plasmid was included to determine and normalize transfection efficiency. Experiments varying in the transfection efficiency above 20% were discarded. All data used derived from experiments in which transfection efficiency was greater than 55%. Experiments with difference in transfection efficiency between different experimental points above 20% were discarded.

### 2.2. Western Blot and Immunoprecipitation

Lysates from transfected MCF-7 cells were separated by SDS-PAGE and immunoblotted as previously described [17]. Antibodies against IRS-1 (sc-560) and *β*-actin were purchased from Santa Cruz Biotechnology, Inc. (Santa Cruz, CA, USA). Antibody against FLAG tag (F7425) was from Sigma Aldrich (Co., St. Louis, MO, USA). Antibodies against AKT, phosphorylated AKT (Ser473), Erk1/2, phosphorylated Erk1/2 (Thr202/Tyr204; D13.14.4E), and phospho-(Ser/Thr) PKA substrate antibody (9621) were from Cell Signaling Technologies (Beverly, MA, USA). Antibody against PI3K-p85*α* was from Upstate Biotechnology, Inc. (Lake Placid, NY, USA). For immunoprecipitation, cell lysates (1 mg) were precleared with 1 *μ*g of Normal IgG and then incubated with 4 *μ*g of antibodies against p85*α* ^PI3K^ (SC-1637 Santa Cruz Biotechnology, Inc Santa Cruz, CA, USA) or against FLAG tag (A4596 Sigma Aldrich, Co., St. Louis, MO, USA). Immunocomplexes were precipitated following the manufacturer's instructions and processed for Western blot analysis as described. Image analysis for all gels was performed with ImageJ software using the “Gel Plot” plug-in.

### 2.3. Confocal Laser Scanning Microscopy

MCF-7 cells expressing p85*α* ^PI3K^ WT or its mutants were seeded on 12 mm glass coverslips, with serum starved for 6 h and treated with 10 nM insulin for 10 and 20 minutes. As internal control MCF-7 cell lines were treated for 30 minutes with FBS. After incubation cells were fixed for 20 min with paraformaldehyde (3%, w/v in PBS), permeabilized for 20 min with Triton X-100 (0.2%, v/v in PBS), and incubated for 1 h with PBS containing FCS (1%, v/v). Coverslips were stained by incubation with anti*-*FLAG and anti*-*IRS-1 antibodies diluted 1 : 1,000 in PBS for 3 h followed by three washings with PBS. Coverslips were then incubated with Alexa*-*Fluor 488 anti*-*rabbit 1 : 1,000 and Alexa-Fluor 633 anti*-*mouse 1 : 1,000 in PBS for 1 h. Cells stained with Alexa*-*Fluor 488 anti*-*rabbit and Alexa*-*Fluor 633 anti*-*mouse alone were used for the background control (Figure S1A in Supplementary Material available online at http://dx.doi.org/10.1155/2014/565839). All coverslips were washed three times in PBS, incubated for 10 min with PBS containing Hoechst 33258 (Sigma) at a final concentration of 1 mg/mL, and finally washed three times with PBS. The coverslips were mounted in Mowiol (Calbiochem, CA) on glass slides. All images were captured with Zeiss confocal microscope 510. The microphotographs were analyzed for the colocalization with ImageJ software using the colocalization finder. The JACoP plug-insand was used for the Van Steensel's cross-correlation function (CCF) (Figure S1B) and Pearson's Coefficient (Figure 2) [30]. The fluorophores were imaged separately to ensure no excitation/emission wavelength overlap. For the transfection efficiency, cells were stained with anti‐FLAG primary antibody and Alexa*-*Fluor 488 anti*-*mouse antibody as previously described and analysed by FACS.

### 2.4. Clonogenic Assay

Clonogenic assay was performed as elsewhere described [31]. MCF-7 cells (3 × 10^2^) expressing p85*α* ^PI3K^ WT or its mutants were resuspended in DMEM supplemented or not with 10 nM insulin and seeded into 6 well plates and cultured for 15 days in 5 nM insulin until cells have formed sufficiently large clones (at least 50 cells). Clones were counted after 30′ fixing with a mixture of 6% glutaraldehyde and 0.5% crystal violet with ImageJ software using the “analyze particles” routine.

### 2.5. Cell Growth Analysis

Proliferation was assessed by cell counting and by MTT assays using the 3-(4,5-dimethylthiazol-2-yl)-2,5-biphenyltetrazolium bromide (MTT) (Sigma-Aldrich, Co., St. Louis, MO, USA) as previously described [17].

### 2.6. Cell Cycle Analysis

Cell cycle analysis was performed by fluorescence-activated cell sorting (FACS) as elsewhere described [17]. Fluorescence was determined by using the FACScanto II Flow Cytometer (Becton Dickinson, Franklin Lakes, NJ, USA).

### 2.7. Wound Healing Assay

Wound healing assay was performed as elsewhere described [31]. Confluent MCF-7 cells were scratched with a 200 *μ*L pipette tip, and cellular debris has been removed by washing gently. Following wounding, culture medium was replaced with fresh medium containing 0.5% FCS and cells were exposed to insulin 10 nM for 12 h. Images were acquired on a phase contrast microscope Axio Observer (Carl Zeiss, Inc., Oberkochen, Germany) and wound diameter was measured at 0 and 12 h using the computing software Axio Vision (Carl Zeiss, Inc.).

### 2.8. Statistical Analyses

All data are presented as the means ± S.D. of at least three experiments in triplicates (*n* ≥ 9). Statistical significance between groups was determined using Student's *t*-test (matched pairs test or unmatched test were used as indicated in the figure legends). All statistical analyses have been performed using JMP Software purchased by Statistical Discovery SAS Institute (*P* < 0.05, statistical significance; *P* < 0.001, high statistical significance).

## 3. Results

### 3.1. Role of Phosphorylation of p85*α* ^PI3K^S83 in the Interaction with IRS-1

**Figure 1:**
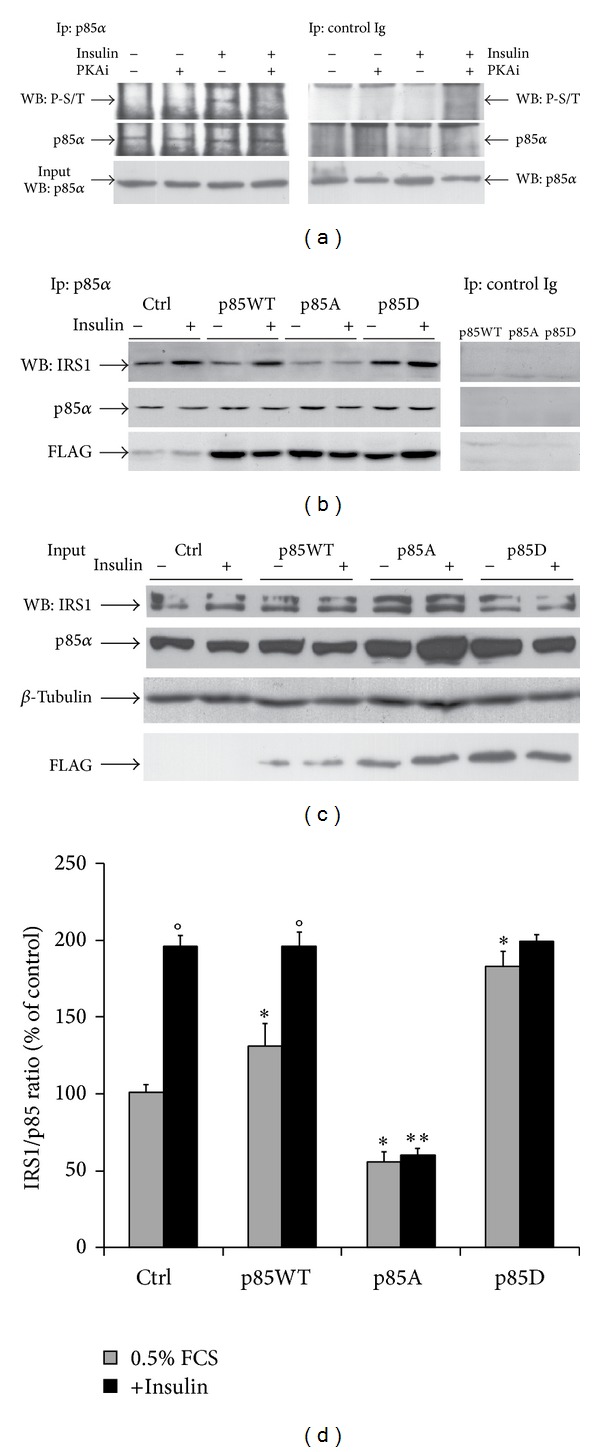
Phosphorylation of p85*α* ^PI3K^S83 modulates the IRS-1/p85 interaction. Total protein extract obtained from MCF-7 cells untreated and treated with 10 nM insulin for 10 min in the pretreated or not with 10 *μ*g/mL PKAi for 15 min was immunoprecipitated with anti-p85*α* antibody and probed with anti-phospho (Ser/Thr) PKA substrate antibody (P-S/T) (a). MCF-7 cells transfected with p85WT or p85A or p85D were serum starved for 6 h and treated with 10 nM insulin for 10 min. Cell lysates were immunoprecipitated with anti-p85 antibody and then analysed by Western blot with anti-IRS-1, anti-p85, and anti-FLAG antibodies (b).   p85*α* ^PI3K^, IRS-1, and *β*-tubulin expression levels in input lysates were shown (c). The upper bands in the insert of IRS-1 are not displaced by the control peptide and are considered nonspecific signals (IRS1-specific bands are indicated by the arrow). The histogram (d) represents the quantitation of IRS-1/p85*α* ^PI3K^ signals ratio obtained from different co-IP experiments. To minimize differences in transfection and/or antibody efficiency, the data shown in the panel (d) were obtained by the comparison of different experiments performed with anti-p85*α* (three experiments), anti-IRS-1 (three experiments), and anti-FLAG (three experiments; see Figure S2). The histograms show the fold variation relative to the untreated control cells of the ratio IRS-1/p85. Values are expressed as mean ± SD (*n* = 7). Differences between treatments were tested for statistical significance using Student's matched pairs *t*-test: °*P* < 0.01 versus 0.5% FCS treated cells; *P < 0.01 versus nontransfected cells; **P < 0.01 versus p85*α* ^PI3K^ wild-type or p85D expressing cells or control cells after insulin treatment.

We have previously identified a serine (at codon 83) in the p85*α* ^PI3K^ molecule that is phosphorylated* in vivo* and* in vitro *by PKA [17]. To demonstrate whether S83 is phosphorylated in PKA-dependent manner following insulin treatment, we have untreated and treated MCF-7 cells with insulin in the presence and in the absence of PKA inhibitor (PKAi) as described in material and methods. Total protein extract was immunoprecipitated with antibodies anti-p85*α* and probed with the phospho-(Ser/Thr) PKA substrate antibody.  Figure 1(a) shows that the S83-p85*α* is slightly phosphorylated in starved cells. Insulin treatment (10 nM) for 10 min greatly increases S83 phosphorylation in p85*α*-PKA-dependent manner because it is prevented by 15 min pretreatment with a selective inhibitor of PKA (PKAi, fragment 14–22). To determine the relevance of this site in the formation of the sequestrating complex IRS-1/p85*α* ^PI3K^ [24], we have substituted Ser83 with alanine (p85A) to prevent phosphorylation or with aspartic acid (p85D) to mimic the phosphorylated residue. As this complex can mediate cAMP-PI3K effects on growth and survival, we set out to determine the formation of IRS-1/p85*α* ^PI3K^ complex in cells expressing wild type p85*α* ^PI3K^ or p85A or p85D. MCF-7 were transfected with p85*α* ^PI3K^ wild type or p85A or p85D and 36 h after transfection, the cells were serum starved for 6 h before 10 min insulin (10 nM). Cell lysates were immunoprecipitated with anti-p85 antibody and then analyzed by Western blot with anti-IRS-1 antibody. In the presence of insulin, p85*α* ^PI3K^ wild type efficiently was found associated in a protein complex with IRS-1. This association was significantly inhibited in cells expressing p85A and stimulated in p85D expressing cells. In the absence of insulin, IRS-1/p85*α* ^PI3K^ complex was barely detectable in the p85A expressing cells. In p85D-expressing cells, however, in absence of insulin a robust association of p85*α* ^PI3K^ with IRS-1 suggests that Ser83 in p85*α* ^PI3K^ cooperates with another insulin-mediated signal to regulate p85*α* ^PI3K^ association with IRS-1 (Figure 1(b)). The interaction specificity was assessed using IgG immunoprecipitates as negative control; p85*α* ^PI3K^, IRS-1, and *β*-tubulin expression levels in input lysates were shown (Figure 1(c)). Similar results were obtained in different immunoprecipition experiments performed with anti-IRS-1 and with anti-FLAG antibodies (Figure S2). Changes in the IRS-1/p85 ratio in cells expressing the p85 mutants and determined by comparing all experiments are summarized in the histogram shown in Figure 1(d). Transfection efficiency has monitored by measuring the GFP expression (see Section 2) and only transfection with efficiency higher than 55% was taken (Figure S3A). These findings collectively suggest that phosphorylation by cAMP-PKA is required for the modulation of interaction between endogenous IRS-1 and p85 in MCF-7 cells.

### 3.2. p85*α* ^PI3K^S83 Phosphorylation Role in the Subcellular Localization of p85*α* and IRS-1

Besides its conventional role, IRS-1 has been found in the nuclear compartment in several cell types, including breast cancer cells and breast tumors [32, 33], where it functions as transcriptional coregulator for RNA polymerases I and II [34].* In vivo* transgenic mouse models of breast cancer showed that loss of IRS-1 enhances breast cancer metastasis, supporting the hypothesis that IRS-1 may have a metastasis suppressor function [35]. In addition nuclear IRS-1 may be a useful marker to predict tamoxifen response in patients with early breast cancer, because a reduction in the nuclear localization of IRS-1 has a negative prognosis. Previous reports demonstrate that IRS-1 is chaperoned to the nucleus by other proteins [31, 33].

**Figure 2:**
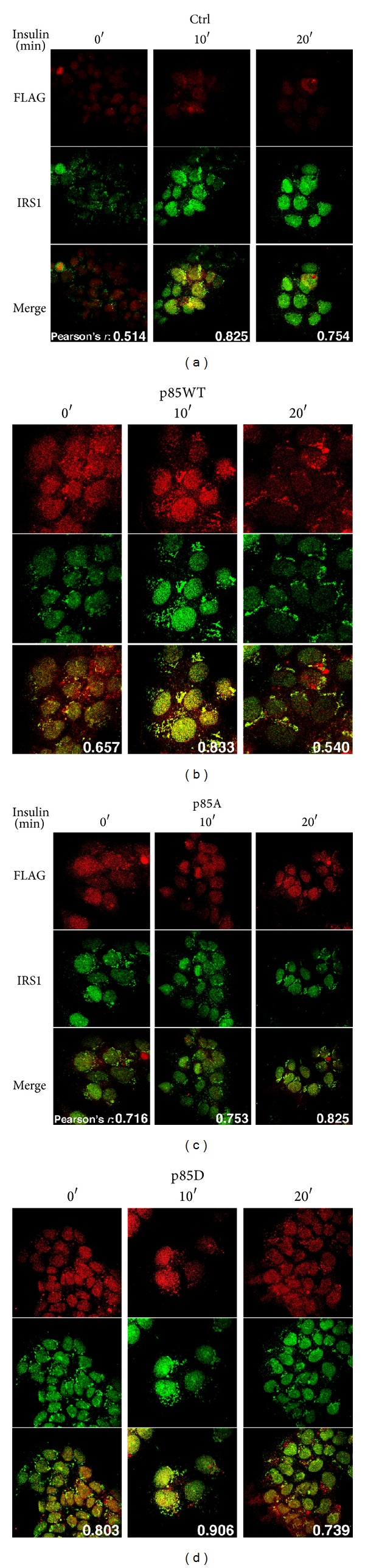
Role of phosphorylation of p85*α* ^PI3K^S83 in the subcellular localization of p85*α* and IRS-1. MCF-7 cells transiently transfected with p85WT or p85A or p85D were seeded on 12 mm glass coverslips, with serum starved for 6 h and treated with 10 nM insulin for 10 and 20 minutes. As internal control cells were treated for 30 minutes with FBS. Immunofluorescence staining was performed as described in Materials and Methods. After incubation cells were fixed, permeabilized, and stained with anti-FLAG and anti-IRS-1 antibodies. Pearson's coefficient is shown in each panel, overlapping images and Van Steensel's cross-correlation functions (CCFs) were calculated for each experimental point (see Figure S1B). Transfection efficiency was determined by flow cytometry using anti-FLAG antibodies (see Section 2) and is shown in Figure S3B.

In order to assess the relevance of p85*α* ^PI3K^S83 with the subcellular localization of IRS-1, MCF-7 were cells transiently transfected with a construct containing p85*α* ^PI3K^  wild type or its S83 mutated forms, seeded on 12-mm glass coverslips, with serum starved for 6 h and treated with 10 nM insulin for 10 and 20 minutes (for transfection efficiency, see Figure S3B). Results demonstrate that insulin induces a rapid and transient formation of IRS-1/p85*α* ^PI3K^ colocalization foci at both cytosolic and nuclear levels (Figure 2 and Figure S1B). In MCF-7 cells expressing p85*α* ^PI3K^wild type, cytosolic-golgi as well as nuclear colocalization of IRS-1/p85*α* ^PI3K^ can be observed. IRS-1/p85*α* ^PI3K^ interaction occurs already after 10 minutes and disappears 20 minutes after exposition to insulin. Interestingly, in p85A expressing cells, there is a marked reduction in the formation of cytosolic IRS-1 aggregates and colocalization with p85*α* ^PI3K^ is not observed (Figure 2). Conversely, cell expressing p85D showed both the IRS-1 cytosolic foci and IRS-1/p85*α* ^PI3K^ counteraction in the absence of insulin. Treatment with 10 nM insulin did not increase the foci and the protein-protein complex formation significantly (Figure 2(d)). It is worth noting that the expression of both mutants (p85A or p85D) reduced the nuclear translocation of IRS-1 suggesting that the presence of a serine at codon 83 is essential to the nuclear translocation of IRS-1, and to the IRS-1/p85*α* ^PI3K^ interaction, as that occurring in cells that express the mutant p85D.

### 3.3. p85*α* ^PI3K^S83 Role in Signal Transduction of Insulin

**Figure 3:**
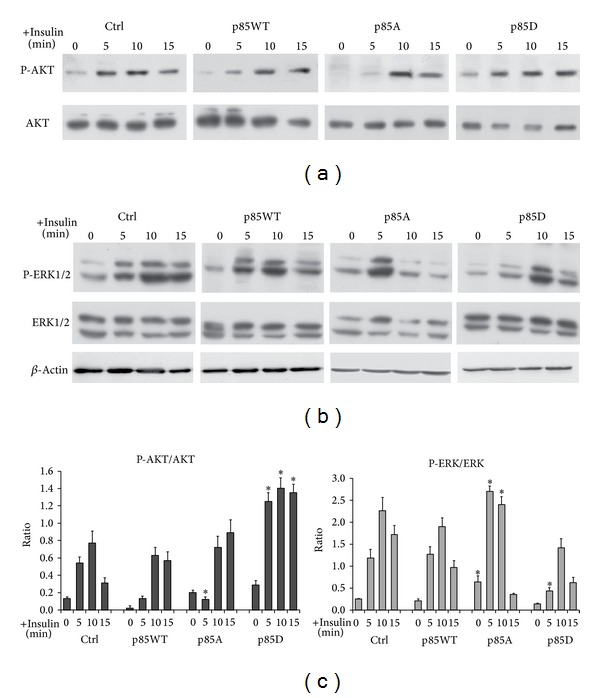
Phosphorylation of p85*α* ^PI3K^S83 modulates postreceptorial transduction. MCF-7 cells were transiently transfected with a construct containing p85WT or its S83 mutated forms and treated with 10 nM insulin for 5, 10, or 15 min. AKT (a) and Erk1/2 (b) phosphorylation were determined through Western blot experiments with antibodies against AKT, phosphorylated AKT (Ser473), Erk1/2, and phosphorylated Erk1/2 (Thr202/Tyr204; D13.14.4E). The histograms in the lower panel (c) show the densitometric analysis of the P-AKT/AKT and P-Erk/Erk ratio relative to *β*-actin, derived from three independent experiments performed in triplicate (*n* = 9). Differences between treatments were tested for statistical significance using Student's matched pairs *t*-test: *P < 0.01 compared to the untransfected cells (same treatment, same time). Transfection efficiency was monitored by measuring the GFP expression (see Section 2) and only transfection with efficiency higher 55% was taken (Figure S3A).

We determined the phosphorylation of AKT and Erk 1/2 in cell lines expressing p85WT or p85*α* mutant in the presence or absence of insulin (10 nM). The P-AKT/total AKT ratio, as well as the ratio P-Erk/total Erk, a rather accurate index of AKT and Erk1/2 activation, was determined. Cells expressing p85*α* ^PI3K^WT, the p85A mutants, or p85D mutants were stimulated with insulin for various periods of time (see Figure 3) and tested for AKT and Erk1/2 phosphorylation. Results show that insulin induced AKT and Erk1/2 phosphorylation and that in cells expressing p85A, AKT induction was delayed and Erk1/2 phosphorylation was anticipated (peak at 15 min instead of 10 minutes for P-AKT; peak at 5 min instead of 10 min for P-Erk) (Figure 3). The expression of p85D did not significantly affect the kinetics of AKT activation by insulin but modified the absolute level of activation (Figures 3(a) and 3(c) left panel). Induction of P-Erk1/2 by insulin was reduced and delayed in cells expressing p85D mutant (Figures 3(b) and 3(c) right panel). These data indicate that serine 83 in p85*α* PI3K is important for insulin induction of AKT and Erk1/2 activation.

### 3.4. Effects of p85*α* Phosphorylation on MCF-7 Cell Proliferation and Viability

Growth is a complex and pleiotropic process, which can be altered by different events. Expression of p85*α* ^PI3K^ driven by a constitutive promoter might induce growth alterations per se. It is well known that PI3K-AKT is one of the most important pathways promoting survival and cell growth [10]. To investigate the role of p85*α* ^PI3K^ mutants in the control of cell proliferation, colorimetric MTT assay and cell cycle analysis by fluorocytometric absorbent cell sorter (FACS) have been performed on MCF-7 cells transiently transfected with p85WT, p85A, or p85D or with the empty vector untreated or treated with insulin (10 nM). Cells were counted at time 0 and at 24, 48, 72, and 96 hours. Colorimetric MTT assay demonstrated that all cells overexpressing p85*α* ^PI3K^ showed an inhibition of cell proliferation, but the growth rate observed in cells overexpressing p85A was lower with respect to that observed in cells overexpressing p85WT or p85D. Insulin treatment had a reduced effect on MCF-7 transfected with p85WT. Overexpression of p85WT negatively regulates the insulin signaling (see Figure 3(c)) leading to a reduced growth response to the hormone. Cells transfected with p85A respond better to insulin treatment because p85A, unable to sequester IRS-1, and induce an earlier and powerful activation of Erk 1/2 (see Figures 3(b) and 3(c)).

**Figure 4:**
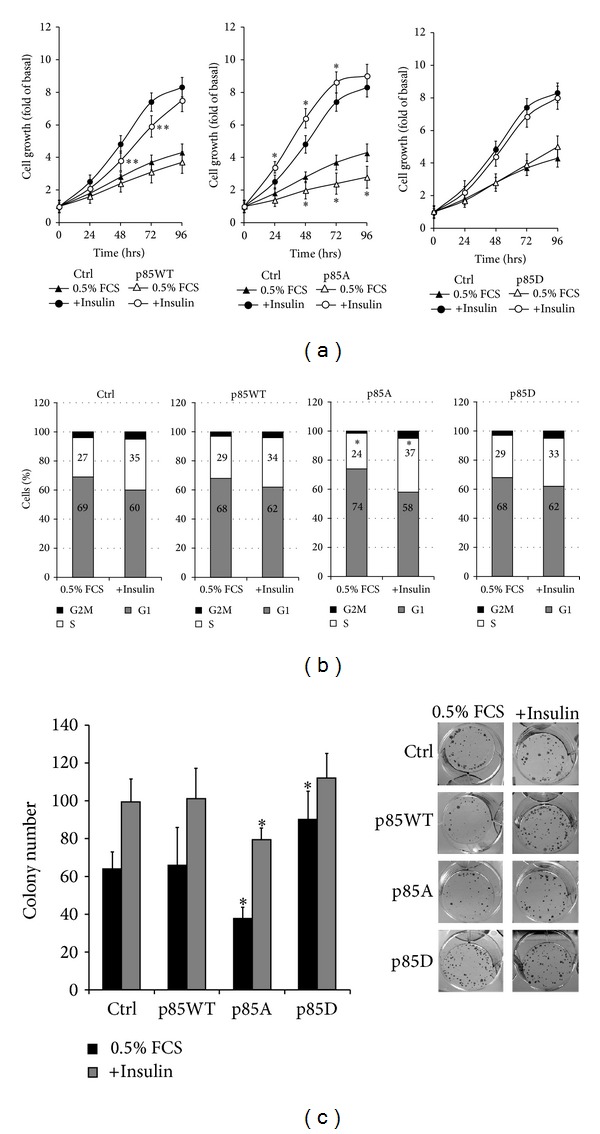
Effects of p85*α* ^PI3K^S83 phosphorylation on MCF-7 cells proliferation and viability. (a) MCF-7 cells transiently transfected with p85WT, p85A, or p85D or with the empty vector were treated with 10 nM insulin or with 0.5% FCS. For the colorimetric MTT assay (A) cells were counted after 0, 24, 48, 72, and 96 hours (**P < 0.01 compared to the control; *P < 0.01 compared to p85WT or p85D). (b) Cell cycle analysis was performed by fluorocytometric absorbent cell sorter (FACS) to determine the percentage of cells in G2 M, S and G1-phase (*P < 0.01 compared to empty vector or p85WT or p85D). The data are the mean of three independent experiments performed in triplicate (*n* = 9). (c) For the clonogenic assay transfected cells were seeded into 6 multiwell plates in the presence of 10 nM insulin. Twenty-four hours later insulin was removed or lowered to 5 nM. Fifteen days later the clones were counted and their cellularity was evaluated by contrast microscopy. Clones were counted after 30′ fixing with a mixture of 6% glutaraldehyde and 0.5% crystal violet. The histogram represents the average number of colonies of four separate experiments performed in duplicate (*n* = 8). (*P < 0.01 versus empty vector).

FACS analysis confirmed that cells expressing p85A showed a lower proliferation rate in basal conditions, as demonstrated by a robust reduction of the percentage of cells accumulated in S and G2, but are more sensitive to insulin treatment (Figure 4(b)).

We also analyzed the ability to form colonies of the cells expressing the wild type and the mutant p85*α* ^PI3K^. 200 cells expressing the various p85*α* ^PI3K^ were seeded in six well plates in the presence of insulin (10 nM). Twenty-four hours later insulin was removed or lowered to 5 nM. Fifteen days later the clones were counted and their cellularity was evaluated by contrast microscopy. Expression of p85A mutant induces the formation of a significant lower number of colonies than the control and p85WT. Cell expressing the p85D mutant formed a large number of clones compared to the control and p85WT. In the presence of 5 nM insulin the control, p85A and p85WT expressing cells increased the number of clones while p85D expressing cells did not do so (Figure 4(c)).

### 3.5. Role of Phosphorylation of p85*α* ^PI3K^S83 in the Control of Motility Behavior

**Figure 5:**
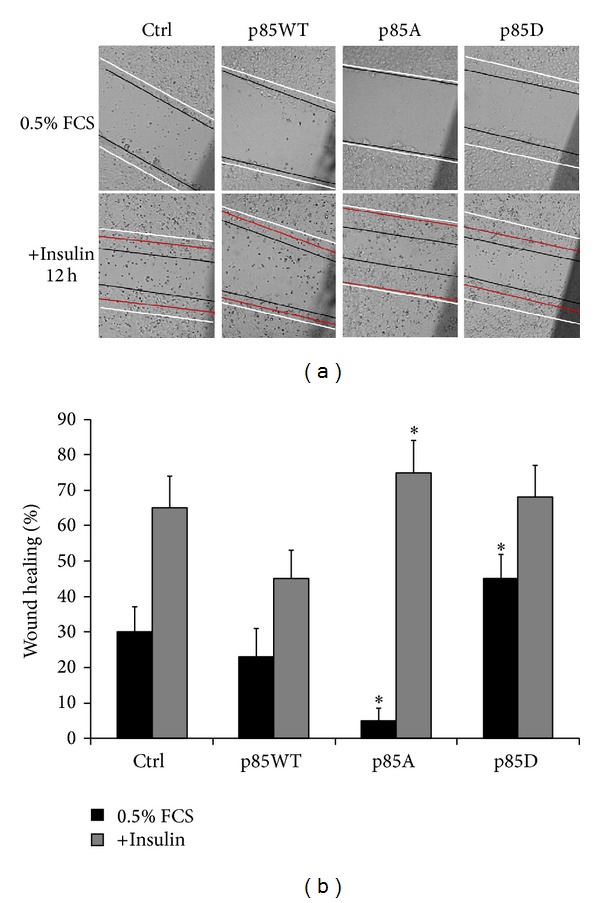
Role of phosphorylation of p85*α* ^PI3K^S83 in the control of migratory behaviour. Wound healing assay on MCF-7 cells overexpressing p85WT or mutants untreated or treated with insulin 10 nM for 12 h. The white lines indicate the limits of the wound; the black lines indicate the limits after 12 h in the presence of 0.5% FCS or in the presence of 10 nM insulin. The red lines in the lower part of the upper panel represent the predicted limits of the wound without insulin. Histogram represents quantification of the insulin treatment effect on cell motility (% of wound healing) calculated as ratio between the space delimited by the white lines and those delimited by the black ones; (*P < 0.01 comparing versus empty vector). Images are representative of three separate experiments performed in triplicate (*n* = 9). These results were confirmed by a transwell migration assay (data not shown).

In many cell types PI3K has multiple roles in cell migration. The role of phosphorylation of p85*α* ^PI3K^S83 in MCF-7 cells' motility was investigated by performing wound healing assays in presence or not of insulin (10 nM). According to our previously published data, the results demonstrated that expression of p85A had an effect* per se *on MCF-7 migration displaying a reduction of cell migration [31]. Insulin treatment induced motility in cells overexpressing empty vector, while it did not influence the motility of cells overexpressing p85WT. The better responsiveness to insulin administration was detectable in cells overexpressing p85A (Figure 5). Our data demonstrated that p85*α* ^PI3K^S83 was essential both for the control of motility behaviour and for the induction of motility induced by insulin.

## 4. Conclusions

Most cancers express both the insulin receptor and the IGF1R genes. At the cellular level, signaling downstream of insulin receptors and hybrid receptors is similar but not identical. In each case, the kinase activity of the receptor leads to phosphorylation of members of the insulin receptor substrate (IRS) family of proteins and this leads to activation of PI3K, AKT, and various downstream networks [36]. However, different cell types use this control system to regulate different processes. For example, two major consequences of pathway activation in liver are the inhibition of gluconeogenesis and the activation of glycogenosynthesis. By contrast, epithelial cells do not express gluconeogenic enzymes and the activation of this pathway leads to the stimulation of proliferation and the inhibition of apoptosis. The data presented herein indicated the relevance of p85*α* ^PI3K^S83 in the response to the activation of insulin receptor pathways. Activation of PI3-kinase in the human breast cancer cell line MCF-7 by insulin-like growth factor-I results in cell cycle progression and tyrosine phosphorylation of IRS-1 [37]. Experimental and clinical data implicate the IGF-IR and PI3K in breast cancer etiology [38]. Recently, frequent occurrence of p85*α* ^PI3K^ mutations in glioblastoma and in colorectal, pancreatic, and breast tumour samples has been reported [39, 40]. However, the ability and role of these mutations in promoting oncogenesis are not still understood. In primary breast tumors, the IGF-IR is overexpressed and hyperphosphorylated, in correlation with unresponsiveness to radiotherapy and tumor relapse [41].

A stable physical interaction between IRS-1 and p85*α* ^PI3K^ has been demonstrated in different cell lines. This study demonstrates that IRS-1 interaction with p85*α* ^PI3K^ depends on the presence of p85*α* ^PI3K^S83 and that the phosphorylation at this residue plays an important role in the fine regulation of the binding. The experiment performed with p85*α* mutants suggests that p85*α* ^PI3K^ phosphorylated at S83 by PKA forms a complex with the IRS-1. Indeed, the substitution of Ser83 with alanine (p85A), which prevents the phosphorylation by PKA, abolishes the ability of p85*α* ^PI3K^ to form a complex with IRS-1 both in basal conditions and after stimulation with insulin. Insulin treatment induces the IRS-1 phosphorylation resulting in the binding between IRS-1 and p85*α* ^PI3K^. According to a previous report [29], the presence of an excess of p85 reduces insulin signaling by removing IRS-1 from p85-p110-IRS-1 trimers. In MCF-7, the expression of a recombinant p85*α* ^PI3K^ reduces the insulin signal by attenuating Erk1/2 activation and delaying the activation of AKT. Conversely, the p85 mutants modify the insulin response in different extent. We hypothesize that p85A is unable to bind IRS-1 and, therefore, make the cells more sensitive to RAS-ERK-MAPK pathway activation by insulin. In p85D expressing cells, the constitutive IRS-1/p85D interaction partially increases the insulin sensitivity due to the ability of the p85D mutant to bind and activate the p110^PI3K^ catalytic subunit [15] but shifts the insulin response to PI3K-AKT pathway.

IRS-1 and p85*α* form a complex influencing the insulin response and their subcellular localization [42]. The data presented here demonstrate that, in MCF-7 cells expressing p85*α* ^PI3K^, insulin induces the colocalization of IRS-1 and p85*α* ^PI3K^ and the formation of IRS-1 cytosolic foci in a time dependent manner. The serine at codon 83 has a relevant effect on the formation of the IRS-1/p85*α* ^PI3K^ complex as demonstrated by the reduced ability of the p85A and the high efficiency of the p85D mutant to induce an insulin-mediated IRS-1/p85*α* ^PI3K^ colocalization of IRS-1 and the formation of cytosolic foci.

Recent studies highlighted the importance of p85*α* ^PI3K^S83 in the control of cell growth in different cellular models, such as FRTL-5 [17], NIH3T3 [16], LNCaP cells [43], and vascular SMCs [44], but it is worth noting that the same phosphorylation leads to opposing phenotypes, depending on the cell type. In fact the phosphorylation of p85*α* ^PI3K^S83 inhibits cell proliferation in fibroblasts and VSMC, while it is essential for the correct cell cycle progression in thyroid cells and does not affect cell proliferation of endothelial cells [16, 17, 44]. Our results show that phosphorylation of p85*α* ^PI3K^S83 is essential for MCF-7 cell survival and proliferation, as demonstrated by the low growth rate observed in cells overexpressing the p85A mutant. S83 seems to play a role in mediating the growth effects of insulin. In our model, we demonstrate that the expression of p85*α* ^PI3K^ mutants is responsible for cell cycle progression induced by insulin treatment, due to a significant reduction in the IRS-1/p85*α* ^PI3K^ complex formation.

PI3K isoforms have multiple roles in cell migration in many cell types and systems, but the relative contribution of PI3Ks to different steps of migration depends on the cell state and the combinations of stimuli to which it is exposed [10]. Direct PI3K activation is sufficient to induce cell motility and invasion [45]. To date, the role of p85*α* ^PI3K^S83 in the insulin-regulated migratory behavior of cells has not yet been reported. This study demonstrates that the overexpression of p85A induces a highly significant suppression of MCF-7 cells motility, indicating that the phosphorylation of p85*α* ^PI3K^S83 is a critical regulator of this process. Moreover, our data demonstrate that insulin treatment causes a significant increase of motility only in cells expressing p85A, underlying that S83 is essential also for insulin-mediated migration.

We suggest that serine 83 phosphorylation mediated by cAMP-PKA induces a conformational change in the PI3K complex, resulting in a facilitated binding to insulin receptor and to IRS-1, with modulations of PI3K activity. p85*α* cAMP-PKA mediated phosphorylation is necessary to modulate the insulin response in the MCF-7 cells. S83 has a pivotal role in subcellular localization of p85*α* and IRS1, in the timing of AKT and ERK activation, cell survival, proliferation, and motility. We believe that this site is a nodal point where information from several receptors is channeled to PI3K.

## Supplementary Material

Supplementary Mathods.
Transfection efficiency determined by FACS analysis.
For the immunofluorescence experiment, the MCF7 cells were transiently transfected with p85WT and its mutants, as followed described: 2,5”106 cells were seeded in 100mm plates containing 9 round coverslips (GG-12-gelatin neuVitr0). After 12h, cells were transfected as described in Section 2 and 48h later, the coverslips were picked up and stained for immufluorescence as described in Section2. The remnant cells were harvested and fixed with paraformaldehyde (2% w/v in PBS) for 10 min and ethanol 70% for 20 min; permeabilized with Triton X-100 (0,2% v/v in PBS) for 20 min. Then cells were stained with anti-FLAG antibody diluited 111000 and with Alexa-Fluor 488 anti-mouse 111000 in PBS, and analysed by FACS for the transfection efficiency (see Figure S3B). For all others experiments the MCF7 cells were transiently transfected with p85WT or its mutants in the presence of pEGFPC3 plasmid. After 48h, the cells were harvested and analyzed for transfection efficiency by FACS analysis.
