# Supplementary material for: The p85 Regulatory Subunit of PI3K Mediates cAMP-PKA and Insulin Biological Effects on MCF-7 Cell Growth and Motility
Source: ScientificWorldJournal. 2014 Jul 9;2014:565839. doi: 10.1155/2014/565839 (PMC4119716; doi:10.1155/2014/565839)
Supplement: Supplementary file 1 — Supplementary Mathods. Transfection efficiency determined by FACS analysis. For the immunofluorescence experiment, the MCF7 cells were transiently transfected with p85WT and its mutants, as followed described: 2,5”106 cells were seeded in 100mm plates containing 9 round coverslips (GG-12-gelatin neuVitr0). After 12h, cells were transfected as described in Section 2 and 48h later, the coverslips were picked up and stained for immufluorescence as described in Section2. The remnant cells were harvested and fixed with paraformaldehyde (2% w/v in PBS) for 10 min and ethanol 70% for 20 min; permeabilized with Triton X-100 (0,2% v/v in PBS) for 20 min. Then cells were stained with anti-FLAG antibody diluited 111000 and with Alexa-Fluor 488 anti-mouse 111000 in PBS, and analysed by FACS for the transfection efficiency (see Figure S3B). For all others experiments the MCF7 cells were transiently transfected with p85WT or its mutants in the presence of pEGFPC3 plasmid. After 48h, the cells were harvested and analyzed for transfection efficiency by FACS analysis. [file 565839.f1.pdf]

The p85 regulatory subunit of PI3K mediates cAMP–PKA and insulin biological effects on MCF-7 cell growth and motility

Di Zazzo E.[1], Feola A.[2,3], Zuchegna C.[2], Romano A.[2], Donini C.F.[4], Bartollino S.[1], Costagliola C.[1], Frunzio R.[2], Laccetti P.[2], Di Domenico M.[3,5]*, Porcellini A[2*].

1. Department of Medicine and Health Sciences, University of Molise, Campobasso;
2. Department of Biology, University Federico II of Naples, Naples, Italy;
3. Department of Biochemistry, Biophysics and General Pathology, Second University of Naples, Italy;
4. Department of Experimental Medicine, Sapienza University of Rome, Rome, Italy;
5. Sbarro Institute for Cancer Research and Molecular Medicine, Center for Biotechnology, Temple University, Philadelphia, Pennsylvania.

SUPPLEMENTARY INFORMATIONS

Supplement Index:

Supplementary Methods page 02

Summary of Supplementary Figures page 03

Supplementary Figures S1-S3 pages 04-06

Supplementary Mathods

Transfection efficiency determined by FACS analysis.

For the immunofluorescence experiment, the MCF7 cells were transiently transfected with p85WT and its mutants, as followed described: 2,5^10^6 cells were seeded in 100mm plates containing 9 round coverslips (GG-12-gelatin *neuVitro*). After 12h, cells were transfected as described in Section 2 and 48h later, the coverslips were picked up and stained for immufluorescence as described in Section2. The remnant cells were harvested and fixed with paraformaldehyde (2% w/v in PBS) for 10 min and ethanol 70% for 20 min; permeabilized with Triton X-100 (0,2% v/v in PBS) for 20 min. Then cells were stained with anti-FLAG antibody diluited 1:1000 and with Alexa-Fluor 488 anti-mouse 1:1000 in PBS, and analysed by FACS for the transfection efficiency (see Figure S3B). For all others experiments the MCF7 cells were transiently transfected with p85WT or its mutants in the presence of pEGFPC3 plasmid. After 48h, the cells were harvested and analyzed for transfection efficiency by FACS analysis.

Summary of Supplementary Figures

**Figure S1. Subcellular localization of p85α and IRS-1 in cells expressing the p85α mutants.**
**Figure S2. Role of p85α^{PI3K}S83 phosphorylation in IRS-1/p85α interaction.**
**Figure S3. Transfection efficiency determined by FACS analysis.**

Fig. S1

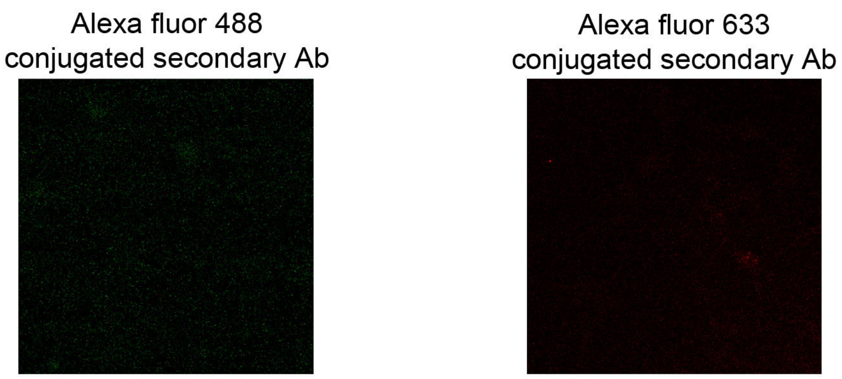

(a)

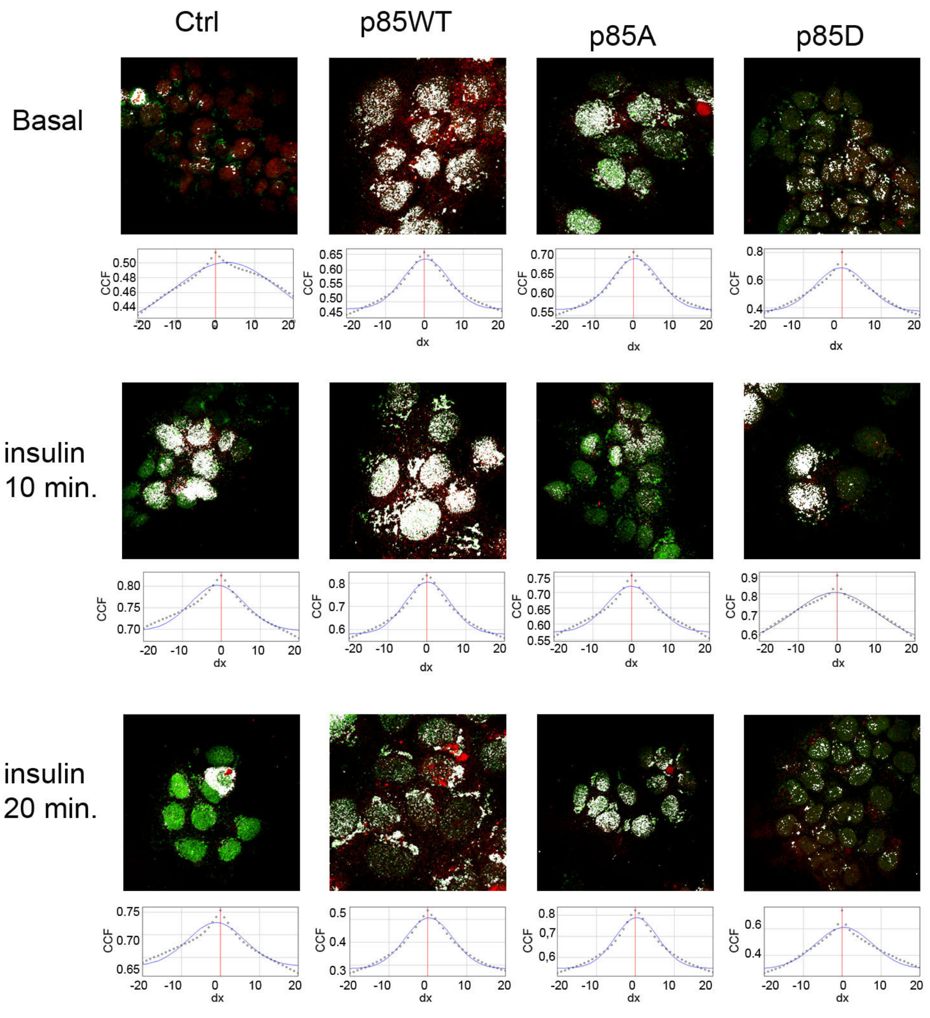

(b)

Figure S1. Subcellular localization of p85α and IRS-1 in cells expressing the p85α mutants.

MCF7 cells transiently transfected with p85WT or p85A or p85D were seeded on 12-mm glass coverslips, serum starved for 6 h and treated with 10 nM insulin for 10 and 20 minutes. As internal control cells were treated for 30 minutes with FBS. Immunofluorescence staining was performed as described in Materials and Methods. All images were captured with Zeiss confocal microscope 510. After incubation cells were fixed, permeabilized and stained with anti-FLAG and anti-IRS1 antibodies. **(a).** Cells stained with Alexa-Fluor 488 anti-rabbit (right panel) and Alexa-Fluor 633 anti-mouse (left panel) alone. **(b).** Colocalization analysis. The microphotographs were analyzed with ImageJ software using the "JACoP" [30] plug-in and the resulting overlapping images (upper panel) are shown. Pearson's coefficient (see Figure 2) and the Van Steensel's Cross-correlation functions (CCFs) (lower panel) were calculated for each experimental point.

Fig. S2

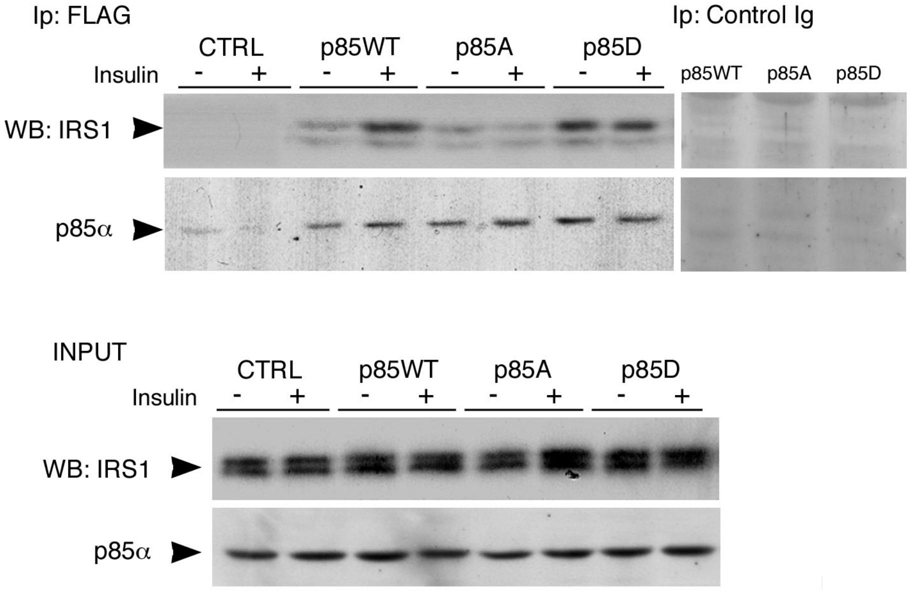

Figure S2. Role of p85α^{PI3K}S83 phosphorylation in IRS-1/p85α interaction.

MCF7 cells transfected with p85WT or p85A or p85D were serum starved for 6 h and treated with 10 nM insulin for 10 min. Cell lysates were immunoprecipitated with anti-FLAG antibody and then analysed by Western blot with anti-IRS-1 and anti-p85α antibodies. In the lower part of the figure is shown the amount of IRS1 and p85α protein present in total protein extract.

Fig. S3

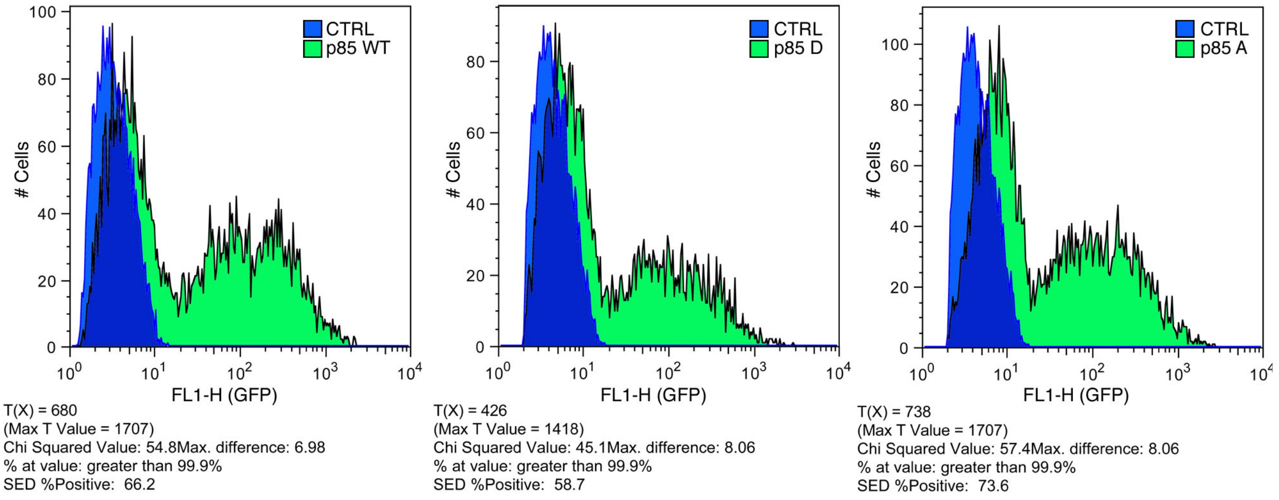

(a)

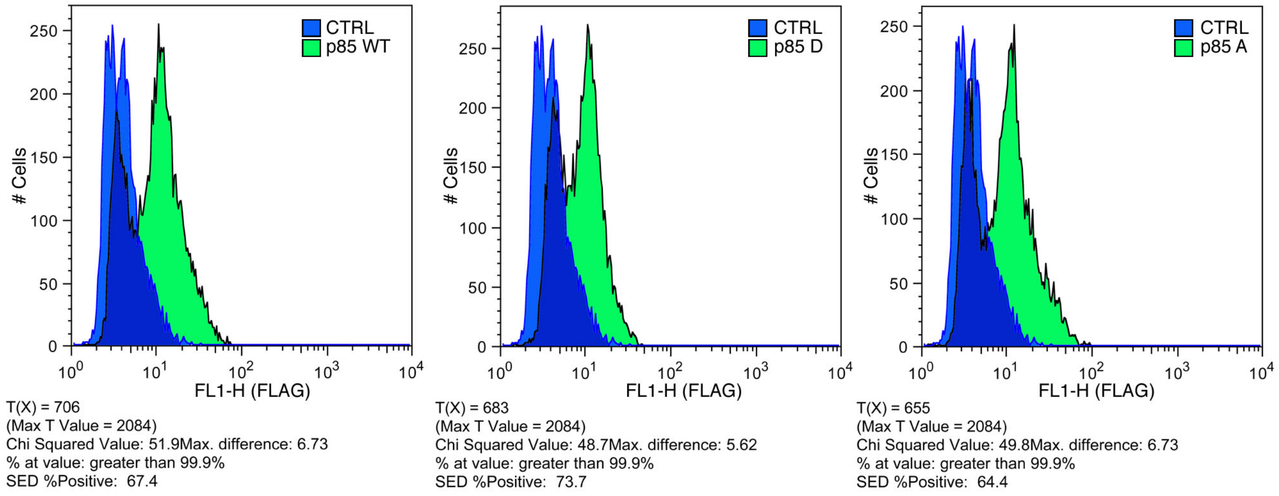

(b)

Figure S3. Transfection efficiency determined by FACS analysis.

The transfection efficiency was measured by FACS analysis using the pEGFPC3 plasmid as reporter. Panel **A** shows a representative experiment. MCF7 cells were transiently transfected with p85WT or p85A or p85D in the presence of pEGFPC3 plasmid. 48h after transfection percentage of GFP positive cells were determined by FACS analysis (**a**). For the confocal analysis, MCF7 cells transiently transfected with p85WT or p85A or p85D, were fixed for 10 minutes with paraformaldehyde (2% w/v in PBS) and for 20 minutes with 70% ethanol, permeabilized for 20 minutes with Triton X-100 (0,2% v/v in PBS) and then stained with anti-FLAG antibody diluted 1:1000 in PBS for 1h (F7425). Cells were incubated with Alexa-Fluor 488 anti-mouse 1:1000 in PBS for 1h (**b**). Blue: untransfected, control MCF-7 cells; Green transfected MCF-7 cells. The percentage of positive cells was calculated using the "Populatin Comparison" tool of the FlowJo Software.
